# The Virtual Metabolic Human database: integrating human and gut microbiome metabolism with nutrition and disease

**DOI:** 10.1093/nar/gky992

**Published:** 2018-10-29

**Authors:** Alberto Noronha, Jennifer Modamio, Yohan Jarosz, Elisabeth Guerard, Nicolas Sompairac, German Preciat, Anna Dröfn Daníelsdóttir, Max Krecke, Diane Merten, Hulda S Haraldsdóttir, Almut Heinken, Laurent Heirendt, Stefanía Magnúsdóttir, Dmitry A Ravcheev, Swagatika Sahoo, Piotr Gawron, Lucia Friscioni, Beatriz Garcia, Mabel Prendergast, Alberto Puente, Mariana Rodrigues, Akansha Roy, Mouss Rouquaya, Luca Wiltgen, Alise Žagare, Elisabeth John, Maren Krueger, Inna Kuperstein, Andrei Zinovyev, Reinhard Schneider, Ronan M T Fleming, Ines Thiele

**Affiliations:** 1 Luxembourg Centre for Systems Biomedicine, University of Luxembourg , Campus Belval, Esch-sur-Alzette L-4367, Luxembourg; 2 Institut Curie, PSL Research University, INSERM U900, F-75005 Paris, France and CBIO-Centre for Computational Biology, MINES ParisTech, PSL Research University, F-75006 Paris, France; 3 Division of Systems Biomedicine and Pharmacology, Leiden Academic Centre for Drug Research, Faculty of Science, University of Leiden, Leiden 2333, The Netherlands

## Abstract

A multitude of factors contribute to complex diseases and can be measured with ‘omics’ methods. Databases facilitate data interpretation for underlying mechanisms. Here, we describe the Virtual Metabolic Human (VMH, www.vmh.life) database encapsulating current knowledge of human metabolism within five interlinked resources ‘Human metabolism’, ‘Gut microbiome’, ‘Disease’, ‘Nutrition’, and ‘ReconMaps’. The VMH captures 5180 unique metabolites, 17 730 unique reactions, 3695 human genes, 255 Mendelian diseases, 818 microbes, 632 685 microbial genes and 8790 food items. The VMH’s unique features are (i) the hosting of the metabolic reconstructions of human and gut microbes amenable for metabolic modeling; (ii) seven human metabolic maps for data visualization; (iii) a nutrition designer; (iv) a user-friendly webpage and application-programming interface to access its content; (v) user feedback option for community engagement and (vi) the connection of its entities to 57 other web resources. The VMH represents a novel, interdisciplinary database for data interpretation and hypothesis generation to the biomedical community.

## INTRODUCTION

Metabolism plays a crucial role in human health and disease, and it is modulated by intrinsic (e.g. genetic) and extrinsic (e.g. diet and gut microbiota) factors. When considered individually, these factors do not sufficiently explain the development and progression of many complex non-communicable diseases, including metabolic syndrome and neurodegenerative diseases. Hence, a systems approach is necessary to elucidate the contribution of each of these factors and to enable the development of efficient, novel treatment strategies.

Such a systems approach requires the easy sharing of knowledge and experimental data generated by different research communities. Databases represent a compelling method of storing, connecting, and making available a vast variety of information derived from primary literature, experimental data, and genome annotations. In fact, biological databases have become valuable tools for facilitating knowledge distribution and enabling research endeavors.

There is a wealth of biochemical databases (1), however, a database that explicitly connects human metabolism with genetics, human-associated microbial metabolism, nutrition, and diseases has not yet been developed. One reason for the lack of such a database may be the use of non-standardized nomenclature, which complicates data integration. Moreover, manual curation of database content is time consuming and requires expert domain knowledge.

Genome-scale metabolic reconstructions represent the full repertoire of known metabolism occurring in a given organism and describe the underlying network of genes, proteins and biochemical reactions (2). High-quality reconstructions go through an intensive manual curation process that follows established protocols to ensure high standards and coverage of the information available on the organism (3). Thus, metabolic reconstructions are valuable knowledge bases that summarize current information on metabolism within organisms. Genome-scale metabolic reconstructions have been generated for representatives of all domains of life, including humans (4) and gut microbes (5–8). Importantly, these metabolic reconstructions can be converted into computational models using condition-specific information, e.g. transcriptomic (9) or metabolomic data (10,11). Open-access, community-developed toolboxes, such as the Constraint-Based Reconstruction and Analysis (COBRA) Toolbox (10), facilitate simulations with metabolic models that permit us to address a variety of biomedical and biotechnological questions *in silico* (12,13).

**Figure 1.**
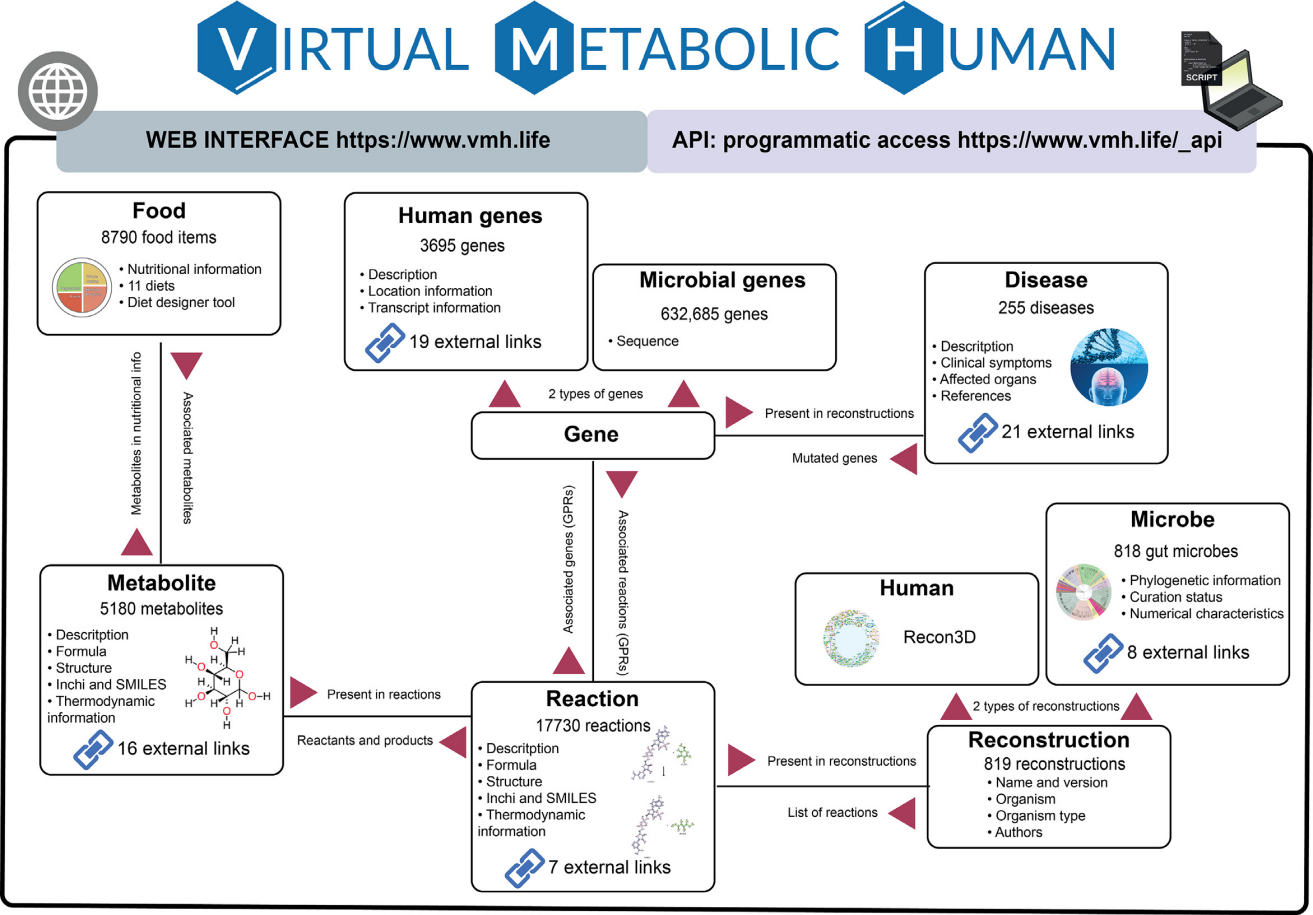
Overview of the Virtual Metabolic Human (VMH) database. The VMH database is divided into two interfaces, and its database contains five distinct but connected resources. Users can interact with the database using the two available interfaces: (i) a user-friendly web interface and (ii) an application-programming interface that allows programmatic access to the information contained in the database. At the core of the database is the representation of reconstructions as sets of reactions. The database connects the five resources through shared nomenclature: (i) the ‘Human metabolism’ and ‘Gut microbiome’ resources share metabolites and reactions, (ii) the nutrients in the ‘Nutrition’ resource are mapped to metabolites that can be shared by the human and gut microbes and (iii) the diseases in the ‘Disease’ resource include affected genes and metabolite biomarkers present in the ‘Human metabolism’ resource. Finally, the ‘ReconMaps’ resource is connected to the ‘Human metabolism’ resource via metabolites and reactions.

Here, we describe the Virtual Metabolic Human (VMH, https://vmh.life) database, which consists of the five interconnected resources: ‘Human metabolism’, ‘Gut microbiome’, ‘Disease’, ‘Nutrition’ and ‘ReconMaps’. These resources are interlinked based on shared nomenclature and database entries for metabolites, reactions and genes (Figure 1). Given the extensively curated, diverse information captured in the VMH database, this resource represents a unique, comprehensive and multi-faceted overview of human and human-associated microbial metabolism.

## DATABASE DESCRIPTION

**Figure 2.**
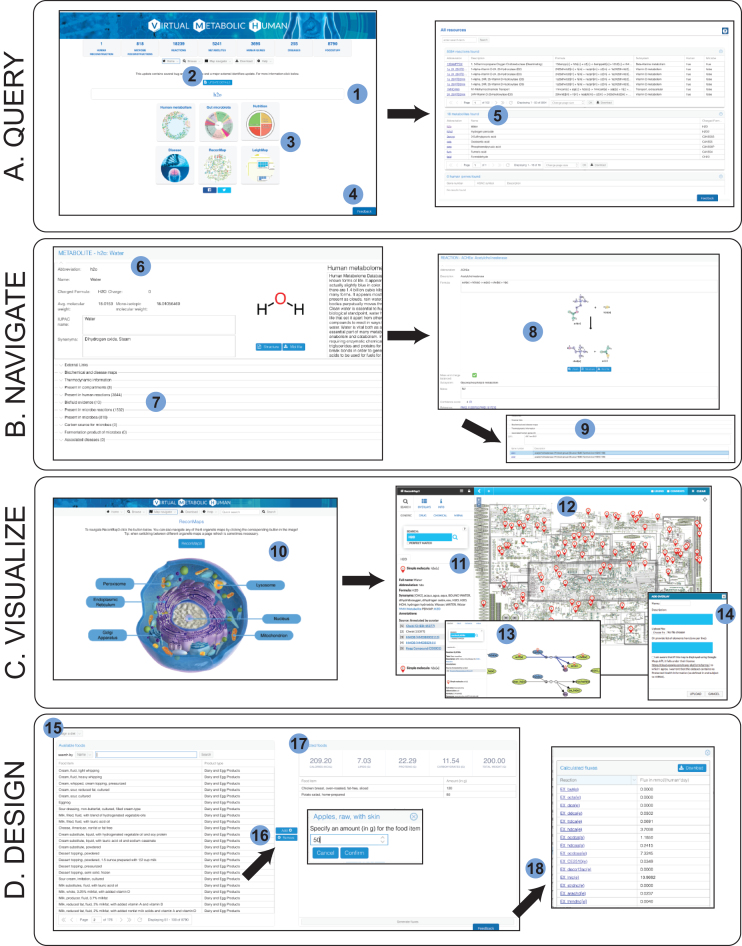
Overview of the VMH functionalities. Users can search all resources, using the Quick Search bar (1), or specific resources through the ‘Browse’ button (2) or the resource panels available in the main page (3). At any point in time, it is possible to provide feedback or report issues with the VMH through the feedback button (4). If a user performs a quick search (e.g. ‘h2o’) different result grids will be available. Each type of entity will be displayed in its corresponding grid (5). Each detail page (6) contains additional information and connections with other resources (both internal and external – 7). For instance, by selecting ‘Associated human reactions’ a user can then navigate to a reaction detail page (8) and from there to other associated entities, such as human genes (9). The VMH also allows the visualization of metabolic pathways through the ‘ReconMaps’ resource (10). Users can search for a metabolite using the side bar of the map interface (11) and get results as locations in the map panel (12). It is also possible to search for specific reactions making it easier to investigate specific pathways of interest (13) and upload simulation or experimental data (14) through the interface or the COBRA Toolbox (10). With the nutrition resource the VMH offers the ability to design *in silico* diets that can be used to perform simulations. In this interface, users can search foods from the ‘Available foods’ panel (15) and add them to the ‘Selected foods’ panel by specifying the portion size in grams (16). During this process, the top of the ‘Selected foods’ panel will automatically update information about the diet (17). When this process is completed, the user can download the flux values to integrate in his experiments (18).

The VMH database contains 17 730 unique reactions, 5180 unique metabolites, 3695 human genes, and 632 685 microbial genes as well as 255 diseases, 818 microbes and 8790 food items. Unique features of the VMH database include (i) metabolic reconstructions of human and gut microbes that can be used as a starting point for simulations; (ii) seven comprehensive maps of human metabolism that permit a visualization of omics data and simulation results; (iii) a nutrition designer that allows researchers to design personal dietary plans for computational simulations; (iv) a user-friendly web interface for browsing, querying and downloading the VMH database content; (v) a well-documented representational state transfer application-programming interface (API) for easy access to the database content; and vi) user feedback integration through the feedback button accessible in all pages of the website and the ReconMaps interface, which allows users to leave comments on specific reactions and metabolites (Figure 2). Great emphasis has been placed on collecting a comprehensive set of database-dependent and independent identifiers to allow for the identification of each entry of the different resources. Additionally, we cross-reference the entries to more than 30 external resources (Table 1), thereby facilitating the access to further metabolic, genetic, clinical, nutritional and toxicological information.

**Table 1.** The connectivity of the VMH: list of external resources connected to the database's entities and the corresponding coverages

Database	Description	URL	Coverage
**Metabolites**			
BIGG	‘BiGG Models is a knowledgebase of genome-scale metabolic network reconstructions. It integrates more than 70 published genome scale networks.’	http://bigg.ucsd.edu/	4670/5180 = 90.2%
Biocyc	‘BioCyc integrates sequenced genomes with predicted metabolic pathways for thousands of organisms and provides extensive bioinformatics tools.’	https://biocyc.org/	863/5180 = 16.7%
ChEBI	‘Chemical Entities of Biological Interest (ChEBI) is a freely available dictionary of molecular entities. The database is focused on ‘small’ chemical compounds, such as atoms, molecules, ions, etc.’	https://www.ebi.ac.uk/chebi/	4770/5180 = 92.1%
Chemspider	‘ChemSpider is a free chemical structure database owned by the Royal Society of Chemistry. It provides fast access to over 60 million structures, properties, and associated information.’	http://www.chemspider.com/	1357/5180 = 26.2%
Drugbank	‘The DrugBank database is a unique bioinformatics and cheminformatics resource that combines detailed drug data with comprehensive drug target information.’	https://www.drugbank.ca/	271/5180 = 5.2%
EPA	‘United States Envrionmental Protection Agency – Chemistry Dashboard.’	https://comptox.epa.gov/dashboard/	793/5180 = 15.3%
Foodb	‘FooDB is a comprehensive resource on food constituents, chemistry and biology. It provides information on both macronutrients and micronutrients.’	http://foodb.ca/	1354/5180 = 26.1%
HMDB	‘The Human Metabolome Database (HMDB) is a freely available database containing detailed information about 114 100 small molecule metabolites found in the human body.’	http://www.hmdb.ca/	5008/5180 = 96.7%
KEGG	‘The Kyoto Encyclopedia of Genes and Genomes integrates genomic, chemical and systemic functional information. KEGG is an integrated database resource consisting of eighteen databases.’	http://www.genome.jp/kegg/	4773/5180 = 92.1%
MetanetX	‘MetaNetX.org is an online platform for accessing, analyzing and manipulating genome-scale metabolic networks and biochemical pathways.’	https://www.metanetx.org/	4989/5180 = 96.3%
METLIN	‘METLIN is a comprehensive MS/MS metabolite database. METLIN includes 961 829 molecules ranging from lipids, steroids, plant & bacteria metabolites, small peptides, carbohydrates, exogenous drugs/metabolites, central carbon metabolites and toxicants.’	https://metlin.scripps.edu/	1372/5180 = 26.5%
ModelSEED	‘ModelSEED is a resource for the reconstruction, exploration, comparison, and analysis of metabolic models.’	http://modelseed.org/	1606/5180 = 31.0%
PDMAP	‘The PD map is a manually curated knowledge repository established to describe molecular mechanisms of PD. It compiles literature-based information on PD into an easy to explore and freely accessible interactive molecular interaction map.’	https://pdmap.uni.lu/MapViewer/	282/5180 = 5.4%
PubChem	‘Pubchem is an open chemistry database that collects information on chemical structures, identifiers, chemical and physical properties, biological activities, patents, health, safety, toxicity data, and others.’	https://pubchem.ncbi.nlm.nih.gov/	4979/5180 = 96.1%
KNApSAcK	‘KNApSAcK is a comprehensive species-metabolite relationship database.’	http://kanaya.naist.jp/KNApSAcK/	446/5180 = 8.6%
Wikipedia	‘Wikipedia is a free online encyclopedia, created and edited by volunteers around the world and hosted by the Wikimedia Foundation.’	https://www.wikipedia.org/	757/5180 = 14.6%
**Reactions**			
BRENDA	‘BRENDA is the main collection of enzyme functional data available to the scientific community.’	http://www.brenda-enzymes.org/	14864/17730 = 83.8%
COG	‘Phylogenetic classification of proteins encoded in complete genomes.’	https://www.ncbi.nlm.nih.gov/COG/	11238/17730 = 63.4%
MetanetX	‘MetaNetX.org is an online platform for accessing, analyzing and manipulating genome-scale metabolic networks and biochemical pathways.’	https://www.metanetx.org/	6302/17730 = 35.5%
ModelSEED	‘ModelSEED is a resource for the reconstruction, exploration, comparison, and analysis of metabolic models.’	http://modelseed.org/	2542/17730 = 14.3%
KEGG Reaction	‘The Kyoto Encyclopedia of Genes and Genomes integrates genomic, chemical and systemic functional information. KEGG is an integrated database resource consisting of eighteen databases.’	http://www.genome.jp/kegg/	14095/17730 = 79.5%
KEGG Orthology	‘The KO (KEGG Orthology) database is a database of molecular functions represented in terms of functional orthologs.’	http://www.genome.jp/kegg/	11238/17730 = 63.4%
Wikipedia	‘Wikipedia is a free online encyclopedia, created and edited by volunteers around the world and hosted by the Wikimedia Foundation.’	https://www.wikipedia.org/	4/17730 = 0.02%
**Human genes**			
ChEMBL	‘ChEMBL is a database of bioactive drug-like small molecules, it contains 2D structures, calculated properties and abstracted bioactivities.’	https://www.ebi.ac.uk/chembl/	3689/3695 = 99.8%
ClinGene	‘ClinGen is a National Institutes of Health (NIH)-funded resource dedicated to building an authoritative central resource that defines the clinical relevance of genes and variants for use in precision medicine and research.’	https://www.clinicalgenome.org/	3695/3695 = 100.0%
DECIPHER	‘DECIPHER (DatabasE of genomiC varIation and Phenotype in Humans using Ensembl Resources) is an interactive web-based database which incorporates a suite of tools designed to aid the interpretation of genomic variants.’	https://decipher.sanger.ac.uk/	3695/3695 = 100.0%
DiseaseMeth	‘The human disease methylation database, DiseaseMeth version 2.0 is a web based resource focused on the aberrant methylomes of human diseases.’	http://bio-bigdata.hrbmu.edu.cn/diseasemeth/index.html	3695/3695 = 100.0%
Ensembl	‘Ensembl is a genome browser for vertebrate genomes that supports research in comparative genomics, evolution, sequence variation and transcriptional regulation.’	http://www.ensembl.org/	3695/3695 = 100.0%
Entrez gene	‘The Entrez Global Query Cross-Database Search System is a federated search engine, or web portal that allows users to search many discrete health sciences databases at the National Center for Biotechnology Information website.’	https://www.ncbi.nlm.nih.gov/gene	3695/3695 = 100.0%
Geno2MP	‘The Geno2MP browser displays the aggregate variants found by exome sequencing data from a wide variety of Mendelian gene discovery projects and enables users to go from Genotypes to Mendelian Phenotypes found in individuals with that genotype.’	http://geno2mp.gs.washington.edu/Geno2MP/#/	3682/3695 = 99.7%
GHR	‘Genetics Home Reference (GHR) is a service of the National Library of Medicine (NLM), which is part of the National Institutes of Health, an agency of the U.S. Department of Health and Human Services.’	https://ghr.nlm.nih.gov/	3688/3695 = 99.8%
GWAS Catalog	‘GWAS Catalog is the NHGRI-EBI Catalog of published genome-wide association studies.’	https://www.ebi.ac.uk/gwas/home	3692/3695 = 99.9%
GWAS Central	‘GWAS Central is a database of summary level findings from genetic association studies, both large and small.’	https://www.gwascentral.org/	3695/3695 = 100.0%
HGNC	‘The Hugo Gene Nomenclature Committee is responsible for approving unique symbols and names for human loci, including protein coding genes, ncRNA genes and pseudogenes, to allow unambiguous scientific communication.’	https://www.genenames.org/	3695/3695 = 100.0%
Human protein Atlas	‘The Human Protein Atlas is a Swedish-based program initiated in 2003 with the aim to map all the human proteins in cells, tissues and organs using integration of various omics technologies, including antibody-based imaging, mass spectrometry-based proteomics, transcriptomics and systems biology.’	https://www.proteinatlas.org/	3695/3695 = 100.0%
LOVD	‘The Leiden Open (source) Variation Database (LOVD) provides a flexible, freely available tool for Gene-centered collection and display of DNA variations.’	http://www.lovd.nl/3.0/home	3695/3695 = 100.0%
OMIM	‘Online Mendelian Inheritance in Man is a continuously updated catalog of human genes and genetic disorders and traits, with a particular focus on the gene-phenotype relationship.’	https://www.omim.org/	3695/3695 = 100.0%
Uniprot	‘UniProt provides the scientific community with a comprehensive, high-quality and freely accessible resource of protein sequence and functional information.’	http://www.uniprot.org/	3695/3695 = 100.0%
WikiGene	‘WikiGenes is a non-profit initiative to provide a global collaborative knowledge base for the life sciences.’	https://www.wikigenes.org/	3695/3695 = 100.0%
Wikipedia	‘Wikipedia is a free online encyclopedia, created and edited by volunteers around the world and hosted by the Wikimedia Foundation.’	https://www.wikipedia.org/	2088/3695 = 56.5%
**Disease**			
1000 genomes	‘A deep catalog of human genetic variation.’	http://phase3browser.1000genomes.org/Homo_sapiens/	255/255 = 100.0%
CellLines		https://www.coriell.org/	241/255 = 94.5%
ClinGene Dosage	‘The Clinical Genome Resource (ClinGen) consortium is curating genes and regions of the genome to assess whether there is evidence to support that these genes/regions are dosage sensitive and should be targeted on a cytogenomic array.’	https://www.ncbi.nlm.nih.gov/projects/dbvar/clingen/	255/255 = 100.0%
ClinicalTrials.gov	‘ClinicalTrials.gov is a database of privately and publicly funded clinical studies conducted around the world.’	https://clinicaltrials.gov/ct2/home	130/255 = 50.9%
ClinVar	‘ClinVar aggregates information about genomic variation and its relationship to human health.’	https://www.ncbi.nlm.nih.gov/clinvar/	255/255 = 100.0%
EuroGenTest	‘EuroGenTest is part of the OrphaNet portal for rare diseases and orphan drugs. EuroGenTest provides information on diagnostic tests able to establish a diagnosis of a rare disease and that need a rare technical competence, or that is the best standard in a given country.’	https://www.orpha.net/consor/cgi-bin/ClinicalLabs_Search.php?lng=EN	228/255 = 89.4%
GARD	‘The Genetic and Rare Diseases Information Center (GARD) provides the public with access to current, reliable, and easy-to-understand information about rare or genetic diseases.’	https://rarediseases.info.nih.gov/	184/255 = 72.2%
Genetic Alliance	‘Disease InfoSearch provides information about diseases and their related support and advocacy networks.’	http://diseaseinfosearch.org	191/255 = 74.9%
Gene Reviews	‘GeneReviews, an international point-of-care resource for busy clinicians, provides clinically relevant and medically actionable information for inherited conditions in a standardized journal-style format, covering diagnosis, management, and genetic counseling for patients and their families. Each chapter in GeneReviews is written by one or more experts on the specific condition or disease and goes through a rigorous editing and peer review process before being published online.’	https://www.ncbi.nlm.nih.gov/books/NBK1116/	88/255 = 34.1%
Geno2MP	‘The Geno2MP browser displays the aggregate variants found by exome sequencing data from a wide variety of Mendelian gene discovery projects and enables users to go from Genotypes to Mendelian Phenotypes found in individuals with that genotype.’	http://geno2mp.gs.washington.edu/Geno2MP/#/	248/255 = 97.2%
GHR	‘Genetics Home Reference (GHR) is a service of the National Library of Medicine (NLM), which is part of the National Institutes of Health, an agency of the U.S. Department of Health and Human Services.’	https://ghr.nlm.nih.gov/	183/255 = 71.8%
GTR	‘The Genetic Testing Registry (GTR) provides a central location for voluntary submission of genetic test information by providers. The scope includes the test's purpose, methodology, validity, evidence of the test's usefulness, and laboratory contacts and credentials.’	https://www.ncbi.nlm.nih.gov/gtr/	241/255 = 94.5%
GWAS Catalog	‘GWAS Catalog is the NHGRI-EBI Catalog of published genome-wide association studies.’	https://www.ebi.ac.uk/gwas/home	187/255 = 73.3%
GWAS Central	‘GWAS Central is a database of summary level findings from genetic association studies, both large and small.’	https://www.gwascentral.org/	253/255 = 99.2%
LOVD	‘The Leiden Open (source) Variation Database (LOVD) provides a flexible, freely available tool for Gene-centered collection and display of DNA variations.’	http://www.lovd.nl/3.0/home	254/255 = 99.6%
MalaCards	‘MalaCards is an integrated database of human maladies and their annotations, modeled on the architecture and richness of the popular GeneCards database of human genes.’	https://www.malacards.org/	206/255 = 80.8%
MGI	‘MGI is the international database resource for the laboratory mouse, providing integrated genetic, genomic, and biological data to facilitate the study of human health and disease.’	http://www.informatics.jax.org/	241/255 = 94.5%
OMIM	‘Online Mendelian Inheritance in Man is a continuously updated catalog of human genes and genetic disorders and traits, with a particular focus on the gene-phenotype relationship.’	https://www.omim.org/	247/255 = 96.9%
OMIM Clinical Symptoms	Synopsis of clinical symptoms.	https://omim.org/clinicalSynopsis/	230/255 = 90.2%
OrphaNet	‘OrphaNet is the portal for rare diseases and orphan drugs.’	https://www.orpha.net/consor/cgi-bin/index.php?lng=EN	223/255 = 87.5%
Wikipedia	‘Wikipedia is a free online encyclopedia, created and edited by volunteers around the world and hosted by the Wikimedia Foundation.’	https://www.wikipedia.org/	201/255 = 78.8%
**Microbe**			
ENA	‘The European Nucleotide Archive (ENA) provides a comprehensive record of the world's nucleotide sequencing information, covering raw sequencing data, sequence assembly information and functional annotation.’	https://www.ebi.ac.uk/ena	806/818 = 98.5%
Ensembl Bacteria	‘Ensembl Bacteria is a browser for bacterial and archaeal genomes.’	http://bacteria.ensembl.org/index.html	808/818 = 98.8%
GOLD	‘Genomes Online Database (GOLD) is a World Wide Web resource for comprehensive access to information regarding genome and metagenome sequencing projects, and their associated metadata, around the world.’	https://gold.jgi.doe.gov/	798/818 = 97.6%
IMG	‘The mission of the Integrated Microbial Genomes & Microbiomes(IMG/M) system is to support the annotation, analysis and distribution of microbial genome and microbiome datasets sequenced at DOE’s Joint Genome Institute (JGI).’	https://img.jgi.doe.gov/	772/818 = 94.4%
KBASE	‘A collaborative, open environment for systems biology of plants, microbes and their communities.’	https://kbase.us/	804/818 = 98.3%
MicrobeWiki	‘MicrobeWiki is a free wiki resource on microbes and microbiology, authored by students at many colleges and universities.’	https://microbewiki.kenyon.edu/index.php/MicrobeWiki	799/818 = 97.7%
NCBI Taxonomy	‘The Taxonomy Database is a curated classification and nomenclature for all of the organisms in the public sequence databases.’	https://www.ncbi.nlm.nih.gov/taxonomy	817/818 = 99.9%
Uniprot	‘UniProt provides the scientific community with a comprehensive, high-quality and freely accessible resource of protein sequence and functional information.’	http://www.uniprot.org/	815/818 = 99.6%

### ‘Human metabolism’ resource

The VMH database hosts the most recent version of the human metabolic network reconstruction, Recon3D (4), which describes the underlying network of 13 543 metabolic reactions distributed across 104 subsystems, 4140 unique metabolites and 3288 genes expressed in at least one human cell. The content of Recon3D has been assembled through an extensive literature review over the past decade by the systems biology community (4,14–16).

Individual pages are dedicated to each reaction, metabolite and gene. These pages contain information on literature-based evidence as well the relations of the page with other entities in the VMH database (Figures 1 and 2). Novel features of Recon3D include molecular structures and atom mappings (4,17), which are visualized on the metabolite and reaction pages, respectively, in addition to thermodynamic information (18).

### ‘ReconMaps’ resource

The ReconMaps resource consists of seven human metabolic maps drawn manually using CellDesigner (19) and hosted within the web service Molecular Interaction NEtwoRks VisuAlization (MINERVA) (20). Six of these maps correspond to the cellular organelles found in human cells: the mitochondrion, nucleus, Golgi apparatus, endoplasmic reticulum, lysosome and peroxisome. On the organelle level, reactions and pathways are drawn based on the defined subsystems, thus allowing the user to perform focused analyses of metabolism occurring in a particular cellular compartment. The seventh map, which is named ReconMap3, accounts for all six organelle maps plus the human metabolic reactions occurring in the cytosol and the extracellular space. Currently, ReconMap3 covers 8151 of the 13 543 (60%) metabolic reactions and 2763 of the 4140 metabolites (67%) captured in Recon3D.

The maps support low-latency content queries and custom dataset visualizations, which are either represented as a text file or automatically uploaded from the COBRA Toolbox (10,21). Tutorials have been developed demonstrating the visualization of data and simulation results onto the ReconMaps (https://opencobra.github.io/cobratoolbox/stable/tutorials/tutorialRemoteVisualisation.html). Users can submit feedback through the map interfaces by right-clicking on specific elements. From each map entity, users can access the corresponding entry in the VMH database and obtain further information from external resources, such as the HMDB (22), KEGG (23) and CHEBI (24). The VMH connects ReconMaps with the Parkinson's disease map, PDMap (25), which visualizes cellular processes known to be involved in Parkinson's disease through the ‘Biochemical and disease maps’ section on the Metabolite page, where possible. We have identified 168 metabolites that are shared between these maps, providing a connection between the general human metabolism and Parkinson's disease related cellular pathways. Similarly, ReconMaps have been connected to the Atlas of Cancer signaling network resource (ACSN) (26), which visualizes pathways known to be deregulated in cancer cells, through shared 252 proteins implicated in 22 functional modules of ACSN and in 51 subsystems of ReconMaps. Further disease maps are currently assembled by the community (27), and we will continue to increase the connectivity of the VMH and the ReconMaps to these valuable resources. The disease map connections with ReconMaps enable for data analysis and visualization beyond metabolism.

### ‘Gut microbiome’ resource

The ‘Gut microbiome’ resource currently contains 818 manually curated genome-scale metabolic reconstructions for microbes (5) commonly found in the human gastrointestinal tract (28) and belonging to 227 genera and 667 species. All microbial reconstructions were based on literature-derived experimental data and comparative genomics. A typical reconstruction contains a mean (standard deviation) of 774 (275) genes, 1218 (249) reactions, and 944 (143) metabolites. We provide detailed information for each strain and reconstruction. Gene, metabolite and reaction content are available in each microbe detail page. In addition, for each microbe, we have compiled a list of metabolites that can be used as carbon sources or that are products of fermentation, including supporting references. Importantly, this resource shares metabolite and reaction nomenclature with the other resources, thus allowing for an integrative analysis of microbial metabolism with host metabolism and nutrition.

### ‘Disease’ resource

The ‘Disease’ resource aims at connecting diseases and their metabolic features. We have, so far, focused on inborn errors of metabolism (IEMs), by linking 255 diseases (29) to the genes present in the ‘Human metabolism’ resource. A total of 288 unique genes and 1872 unique VMH reactions are associated with these IEMs and provide biochemical and genetic descriptions. We have compiled clinical presentations, genotype-phenotype relationships and the affected organ systems for the IEMs from the primary and review literature. Additionally, we connect each entry with up to 21 external resources, thus providing further information on the diseases, genetic testing and ongoing clinical trials. In the future, we envision the expansion of this resource not only by inclusion of more information on included diseases but also with other diseases with metabolic components.

The VMH database also hosts the Leigh Map (30), which represents a computational gene-to-phenotype diagnosis support tool for mitochondrial disorders. The Leigh Map consists of 87 genes and 234 phenotypes expressed in Human Phenotypic Ontology (HPO) terms (31), and they provide sufficient phenotypic and genetic variation to test the network's diagnostic capability. The Leigh Map is a first step in integrating diagnosis tools within the VMH. Further development of this resource will provide a detailed multi-layered overview of the connection between clinical features, genetic mutations and metabolic pathways facilitating better understanding of the underlying mechanisms of complex diseases.

### ‘Nutrition’ resource

The ‘Nutrition’ resource consists of two parts: (i) a food database mapped onto the metabolites present in the VMH and (ii) a diet database listing the nutritional composition of 11 pre-defined diets. The food database was built by integrating the molecular composition information for 8790 food items distributed in 25 food groups obtained from the USDA National Nutrient Database for Standard Reference (32). Of the 150 nutritional constituents, 100 could be mapped onto the metabolites present in the VMH database. Most of the remaining unmapped constituents represent general metabolite classes (e.g. fibers). The resource can be queried based on food items as well as their nutritional constituents.

The diet database contains 11 diets that were formulated based on real-life examples and literature. For instance, an ‘EU diet’ was designed based on information from an Austrian survey (33). The diets consist of a one-day meal plan and include information on the energy content, fatty acids, amino acids, carbohydrates, dietary fibers, vitamins, minerals and trace elements. The composition of each meal is given in appropriate portion sizes. The information for the nutritional composition of each food item and dish was obtained from the ‘Österreichische Nährwerttabelle’ (http://www.oenwt.at/content/naehrwert-suche/). The molecular composition of a diet can be downloaded in grams per person (70 kg) per day or as a flux rate (in millimoles per person per day), which can be directly integrated with the human metabolic model (4,29) using the COBRA Toolbox (10).

### ‘Diet designer’

The ‘Diet designer’ tool allows users to design their diets (Figure 2D). The interface is divided into two lists: ‘Available foods’ and ‘Selected foods’. Users can search and select any of the available 8790 foods and add them to the list of selected foods by specifying a portion size. As the user designs the diet, the overall information is updated for total calories, lipids, proteins, carbohydrates and portion weight. The user can then see and download the corresponding molecular composition and flux values for the uptake rate of metabolite-mapped nutrients. These flux values can be a starting point for modeling host–microbiome interactions but do not take into consideration differences in absorption along the gastro-intestinal tract. It is also worth mentioning that not all nutrient amounts are converted to metabolite amounts due to the lack of detailed molecular composition information of the food items.

### Resources connectedness

We have focused the design of the VMH on the ability to navigate all its resources seamlessly. From any detail page in the VMH, it is possible to access related entities through links in association grids (Figure 2B). In addition, each entity of the database contains a list of links to external resources with different purposes and focus (e.g. chemistry, nutrition and clinical). We continuously verify the integrity of our links and where possible, use the resolving system Identifiers.org (34). Overall, the VMH connects to 57 different external databases (Table 1). This focus on connectedness will continuously increase the amount and the depth of knowledge that can be accessed through the VMH, thereby increasing the database's utility to the scientific community beyond the systems biology community.

### The VMH beyond computational modeling

A growing number of studies link microbial composition with diet and disease (35,36). The generation of novel hypotheses about the functional implications of observed correlations, e.g. between microbial abundances in disease states, is hindered by the lack of online databases to facilitate such work. In particular, the ‘diet designer’ tool in conjunction with computational modeling permits the generation of *in silico* hypotheses that could then be experimentally tested. Moreover, the use of synthetic microbial communities is of great value for hypothesis testing, and the VMH database could facilitate the design of defined microbial communities with specified metabolic capabilities.

### Accessing the API

The VMH API can be reached at https://www.vmh.life/_api. This page displays some of the available resources that can be used to retrieve data. Each of these is reachable through a Uniform Resource Identifier (URI), which provides data in different formats, such as HTML, JSON or flat file format (CSV). For each of these identifiers, additional query parameters can be applied, which allow to further refine the search (e.g. search a metabolite with a given HMDB identifier). All API endpoints and query parameters are detailed at https://www.vmh.life/_api/docs, where users can test the API usage and get code templates, in different programming languages, to integrate access to the VMH in their applications or scripts.

## DATABASE IMPLEMENTATION

The VMH database was implemented with MySQL 5.6 (https://dev.mysql.com/). The front-end is reachable via web browser at https://vmh.life and was developed in Sencha ExtJS 5.1 (https://www.sencha.com/). The API was developed using Python 2.7 via the DJANGO framework and the Django Rest Framework package.

The diagram editor CellDesigner (version 4.4) (19) was used to manually draw the metabolic maps of the ‘ReconMaps’ resource. Continuous quality control was achieved using a dedicated MATLAB (Mathworks, Inc.) code for map correction and manipulation. This code and the corresponding tutorial are freely available in the COBRA Toolbox (10) (https://opencobra.github.io/cobratoolbox/).

## CONCLUSION

The VMH database captures information on human and gut microbial metabolism and links this information to hundreds of diseases and nutritional data. Therefore, the VMH database addresses an increasing need to facilitate rapid analyses and interpretations of complex data arising from large-scale biomedical studies. Unique and distinguishing features of the VMH database are the following three key factors. First, the VMH database is a comprehensive, interdisciplinary database that permits complex queries. Second, the VMH database provides a graphical representation of the ‘Human metabolism’ resource through the ‘ReconMaps’ resource, thus allowing for the analysis of complex, multi-faceted omics data in the context of the biochemical knowledge captured in the VMH database. Third, the VMH database represents a starting point for computational modeling of human and microbial metabolism in healthy and diseased states by providing information and simulation constraints and being fully compatible with the COBRA Toolbox (10). While the front-end of the VMH database permits complex, interdisciplinary queries by the general user, the comprehensive API enables programmers to perform many complex searches on the database content. As such, the VMH database provides a novel research tool by increasing the availability of diverse data along the diet-gut-health axis to the biomedical community.

## DATA AVAILABILITY

The VMH database and its content are freely available at https://www.vmh.life. Metabolic reconstructions and additional materials are available in the ‘Download’ section, and search results are directly downloadable from the grid interfaces. Users can provide feedback through the different platforms on the website. Detected issues will be addressed and integrated into the database in subsequent releases. The API can be accessed by third-party applications and is also accessible via web browser at https://www.vmh.life/_api. Detailed documentation for the API is available at https://www.vmh.life/_api/docs.

## References

[1] Rigden D.J., Fernandez X.M. The 2018 Nucleic Acids Research database issue and the online molecular biology database collection. Nucleic Acids Res. 2018; 46:D1–D7. 29316735 10.1093/nar/gkx1235 PMC5753253

[2] Palsson B.O. Systems Biology: Properties of Reconstructed Networks. 2006; Cambridge University Press.

[3] Thiele I., Palsson B.O. A protocol for generating a high-quality genome-scale metabolic reconstruction. Nat. Protoc. 2010; 5:93–121. 20057383 10.1038/nprot.2009.203 PMC3125167

[4] Brunk E., Sahoo S., Zielinski D.C., Altunkaya A., Drager A., Mih N., Gatto F., Nilsson A., Preciat Gonzalez G.A., Aurich M.K. et al. Recon3D enables a three-dimensional view of gene variation in human metabolism. Nat. Biotechnol. 2018; 36:272–281. 29457794 10.1038/nbt.4072 PMC5840010

[5] Magnusdottir S., Heinken A., Kutt L., Ravcheev D.A., Bauer E., Noronha A., Greenhalgh K., Jager C., Baginska J., Wilmes P. et al. Generation of genome-scale metabolic reconstructions for 773 members of the human gut microbiota. Nat. Biotechnol. 2017; 35:81–89. 27893703 10.1038/nbt.3703

[6] Heinken A., Khan M.T., Paglia G., Rodionov D.A., Harmsen H.J., Thiele I. Functional metabolic map of Faecalibacterium prausnitzii, a beneficial human gut microbe. J. Bacteriol. 2014; 196:3289–3302. 25002542 10.1128/JB.01780-14 PMC4135701

[7] Shoaie S., Karlsson F., Mardinoglu A., Nookaew I., Bordel S., Nielsen J. Understanding the interactions between bacteria in the human gut through metabolic modeling. Sci. Rep. 2013; 3:2532. 23982459 10.1038/srep02532 PMC3755282

[8] Heinken A., Thiele I. Systematic prediction of health-relevant human-microbial co-metabolism through a computational framework. Gut Microbes. 2015; 6:120–130. 25901891 10.1080/19490976.2015.1023494 PMC4615372

[9] Yizhak K., Gaude E., Le Devedec S., Waldman Y.Y., Stein G.Y., van de Water B., Frezza C., Ruppin E. Phenotype-based cell-specific metabolic modeling reveals metabolic liabilities of cancer. eLife. 2014; 3:e03641. 10.7554/eLife.03641 PMC4238051 25415239

[10] Heirendt L., Arreckx S., Pfau T., Mendoza S.N., Richelle A., Heinken A., Haraldsdottir H.S., Keating S.M., Vlasov V., Wachowiak J. et al. Creation and analysis of biochemical constraint-based models: the COBRA Toolbox v3.0. Nat. Protoc. 2018; https://arxiv.org/abs/1710.04038. 10.1038/s41596-018-0098-2 PMC6635304 30787451

[11] Aurich M.K., Fleming R.M., Thiele I. MetaboTools: a comprehensive toolbox for analysis of Genome-Scale metabolic models. Frontiers in Physiology. 2016; 7:327. 27536246 10.3389/fphys.2016.00327 PMC4971542

[12] Nielsen J. Systems biology of Metabolism: a driver for developing personalized and precision medicine. Cell Metab. 2017; 25:572–579. 28273479 10.1016/j.cmet.2017.02.002

[13] Zhang C., Hua Q. Applications of Genome-Scale metabolic models in biotechnology and systems medicine. Front. Physiol. 2015; 6:413. 26779040 10.3389/fphys.2015.00413 PMC4703781

[14] Thiele I., Swainston N., Fleming R.M., Hoppe A., Sahoo S., Aurich M.K., Haraldsdottir H., Mo M.L., Rolfsson O., Stobbe M.D. et al. A community-driven global reconstruction of human metabolism. Nat. Biotechnol. 2013; 31:419–425. 23455439 10.1038/nbt.2488 PMC3856361

[15] Duarte N.C., Becker S.A., Jamshidi N., Thiele I., Mo M.L., Vo T.D., Srivas R., Palsson B.O. Global reconstruction of the human metabolic network based on genomic and bibliomic data. PNAS. 2007; 104:1777–1782. 17267599 10.1073/pnas.0610772104 PMC1794290

[16] Swainston N., Smallbone K., Hefzi H., Dobson P.D., Brewer J., Hanscho M., Zielinski D.C., Ang K.S., Gardiner N.J., Gutierrez J.M. et al. Recon 2.2: from reconstruction to model of human metabolism. Metabolomics. 2016; 12:109. 27358602 10.1007/s11306-016-1051-4 PMC4896983

[17] Gonzalez G.A.P., El Assal L.R., Noronha A., Thiele I., Haraldsdóttir H.S., Fleming R.M. Comparative evaluation of atom mapping algorithms for balanced metabolic reactions: application to Recon 3D. J. Cheminformatics. 2017; 9:39. 10.1186/s13321-017-0223-1 PMC5471289 29086112

[18] Noor E., Haraldsdottir H.S., Milo R., Fleming R.M. Consistent estimation of Gibbs energy using component contributions. PLoS Comput. Biol. 2013; 9:e1003098. 23874165 10.1371/journal.pcbi.1003098 PMC3708888

[19] Funahashi A., Matsuoka Y., Jouraku A., Morohashi M., Kikuchi N., Kitano H. CellDesigner 3.5: A versatile modeling tool for biochemical networks. Proc. IEEE. 2008; 96:1254–1265.

[20] Gawron P., Ostaszewski M., Satagopam V., Gebel S., Mazein A., Kuzma M., Zorzan S., McGee F., Otjacques B., Balling R. et al. MINERVA-a platform for visualization and curation of molecular interaction networks. NPJ Syst. Biol. Appl. 2016; 2:16020. 28725475 10.1038/npjsba.2016.20 PMC5516855

[21] Noronha A., Danielsdottir A.D., Gawron P., Johannsson F., Jonsdottir S., Jarlsson S., Gunnarsson J.P., Brynjolfsson S., Schneider R., Thiele I. et al. ReconMap: an interactive visualization of human metabolism. Bioinformatics. 2017; 33:605–607. 27993782 10.1093/bioinformatics/btw667 PMC5408809

[22] Wishart D.S., Jewison T., Guo A.C., Wilson M., Knox C., Liu Y., Djoumbou Y., Mandal R., Aziat F., Dong E. et al. HMDB 3.0–The Human Metabolome Database in 2013. Nucleic Acids Res. 2013; 41:D801–D807. 23161693 10.1093/nar/gks1065 PMC3531200

[23] Kanehisa M., Goto S., Sato Y., Furumichi M., Tanabe M. KEGG for integration and interpretation of large-scale molecular data sets. Nucleic Acids Res. 2012; 40:D109–D114. 22080510 10.1093/nar/gkr988 PMC3245020

[24] Hastings J., Owen G., Dekker A., Ennis M., Kale N., Muthukrishnan V., Turner S., Swainston N., Mendes P., Steinbeck C. ChEBI in 2016: Improved services and an expanding collection of metabolites. Nucleic Acids Res. 2016; 44:D1214–D1219. 26467479 10.1093/nar/gkv1031 PMC4702775

[25] Fujita K.A., Ostaszewski M., Matsuoka Y., Ghosh S., Glaab E., Trefois C., Crespo I., Perumal T.M., Jurkowski W., Antony P.M. Integrating pathways of Parkinson's disease in a molecular interaction map. Mol. Neurobiol. 2014; 49:88–102. 23832570 10.1007/s12035-013-8489-4 PMC4153395

[26] Kuperstein I., Bonnet E., Nguyen H.A., Cohen D., Viara E., Grieco L., Fourquet S., Calzone L., Russo C., Kondratova M. et al. Atlas of Cancer Signalling Network: a systems biology resource for integrative analysis of cancer data with Google Maps. Oncogenesis. 2015; 4:e160. 26192618 10.1038/oncsis.2015.19 PMC4521180

[27] Ostaszewski M., Gebel S., Kuperstein I., Mazein A., Zinovyev A., Dogrusoz U., Hasenauer J., Fleming R.M.T., Le Novere N., Gawron P. et al. Community-driven roadmap for integrated disease maps. Brief. Bioinform. 2018; doi:10.1093/bib/bby024. 10.1093/bib/bby024 PMC6556900 29688273

[28] Qin J., Li R., Raes J., Arumugam M., Burgdorf K.S., Manichanh C., Nielsen T., Pons N., Levenez F., Yamada T. et al. A human gut microbial gene catalogue established by metagenomic sequencing. Nature. 2010; 464:59–65. 20203603 10.1038/nature08821 PMC3779803

[29] Sahoo S., Franzson L., Jonsson J.J., Thiele I. A compendium of inborn errors of metabolism mapped onto the human metabolic network. Mol. Biosyst. 2012; 8:2545–2558. 22699794 10.1039/c2mb25075f

[30] Rahman J., Noronha A., Thiele I., Rahman S. Leigh map: a novel computational diagnostic resource for mitochondrial disease. Ann. Neurol. 2017; 81:9–16. 27977873 10.1002/ana.24835 PMC5347854

[31] Köhler S., Doelken S.C., Mungall C.J., Bauer S., Firth H.V., Bailleul-Forestier I., Black G., Brown D.L., Brudno M., Campbell J. The Human Phenotype Ontology project: linking molecular biology and disease through phenotype data. Nucleic Acids Res. 2014; 42:D966–D974. 24217912 10.1093/nar/gkt1026 PMC3965098

[32] US Department of Agriculture, A.R.S. Nutrient Data Laboratory. 2016.

[33] Elmadfa I. Österreichischer Ernährungsbericht 2012. 2012; 1st edn Vienna.

[34] Wimalaratne S.M., Bolleman J., Juty N., Katayama T., Dumontier M., Redaschi N., Le Novere N., Hermjakob H., Laibe C. SPARQL-enabled identifier conversion with Identifiers.org. Bioinformatics. 2015; 31:1875–1877. 25638809 10.1093/bioinformatics/btv064 PMC4443684

[35] Claesson M.J., Jeffery I.B., Conde S., Power S.E., O’Connor E.M., Cusack S., Harris H.M., Coakley M., Lakshminarayanan B., O'Sullivan O. et al. Gut microbiota composition correlates with diet and health in the elderly. Nature. 2012; 488:178–184. 22797518 10.1038/nature11319

[36] Zeevi D., Korem T., Zmora N., Israeli D., Rothschild D., Weinberger A., Ben-Yacov O., Lador D., Avnit-Sagi T., Lotan-Pompan M. et al. Personalized nutrition by prediction of glycemic responses. Cell. 2015; 163:1079–1094. 26590418 10.1016/j.cell.2015.11.001

